# Reduced Mortality among COVID-19 ICU Patients after Treatment with HemoClear Convalescent Plasma in Suriname

**DOI:** 10.1128/mbio.03379-22

**Published:** 2023-02-23

**Authors:** R. Bihariesingh-Sanchit, R. Bansie, N. Ramdhani, R. Mangroo, D. Bustamente, E. Diaz, C. Fung A Foek, I. Thakoer, S. Vreden, Z. Choudhry, A. B. van ‘t Wout, D. A. Diavatopoulos, A. P. Nierich

**Affiliations:** a Department of Anesthesiology, Academic Hospital Paramaribo, Paramaribo, Suriname; b Department of Intensive Care, Academic Hospital Paramaribo, Paramaribo, Suriname; c Department of Internal Medicine, Academic Hospital Paramaribo, Paramaribo, Suriname; d Department of Radiology, Academic Hospital Paramaribo, Paramaribo, Suriname; e Department of Nephrology and Therapeutic Apheresis, Horacio Oduber Hospital, Oranjestad, Aruba; f AlphaBiomics Limited, London, United Kingdom; g van ‘t Wout Pharma Consulting, Amsterdam, The Netherlands; h Section Paediatric Infectious Diseases, Laboratory of Medical Immunology, Radboud Institute for Molecular Life Sciences, Radboud University Medical Center, Nijmegen, The Netherlands; i Radboud Center for Infectious Diseases, Radboud University Medical Center, Nijmegen, The Netherlands; j Departments of Anesthesiology and Intensive Care, Isala, Zwolle, The Netherlands; k HemoClear BV, Zwolle, The Netherlands; Johns Hopkins Bloomberg School of Public Health

**Keywords:** convalescent plasma, COVID-19, HemoClear, ICU, mortality, SARS-CoV-2

## Abstract

Convalescent plasma is a promising therapy for coronavirus disease 2019 (COVID-19), but its efficacy in intensive care unit (ICU) patients in low- and middle-income country settings such as Suriname is unknown. Bedside plasma separation using the HemoClear device made convalescent plasma therapy accessible as a treatment option in Suriname. Two hundred patients with severe SARS-CoV-2 infection requiring intensive care were recruited. Fifty eight patients (29%) received COVID-19 convalescent plasma (CCP) treatment in addition to standard of care (SOC). The CCP treatment and SOC groups were matched by age, sex, and disease severity scores. Mortality in the CCP treatment group was significantly lower than that in the SOC group (21% versus 39%; Fisher’s exact test *P* = 0.0133). Multivariate analysis using ICU days showed that CCP treatment reduced mortality (hazard ratio [HR], 0.35; 95% confidence interval [CI], 0.18 to 0.66; *P* = 0.001), while complication of acute renal failure (creatinine levels, >110 mol/L; HR, 4.45; 95% CI, 2.54 to 7.80; *P* < 0.0001) was independently associated with death. Decrease in chest X-ray score in the CCP treatment group (median −3 points, interquartile range [IQR] −4 to −1) was significantly greater than that in the SOC group (median −1 point, IQR −3 to 1, Mann-Whitney test *P* = 0.0004). Improvement in the PaO_2_/FiO_2_ ratio was also significantly greater in the CCP treatment group (median 83, IQR 8 to 140) than in the SOC group (median 35, IQR −3 to 92, Mann-Whitney *P* = 0.0234). Further research is needed for HemoClear-produced CCP as a therapy for SARS-CoV-2 infection together with adequately powered, randomized controlled trials.

## INTRODUCTION

As of 28 November 2022, the COVID-19 pandemic has resulted in 637 million confirmed cases and 6.6 million deaths globally (WHO, https://covid19.who.int/) since the discovery of severe acute respiratory syndrome coronavirus 2 (SARS-CoV-2) in December 2019 (1, 2). Clinical manifestation ranges from mild upper respiratory tract illness to a diffuse viral pneumonia causing acute respiratory failure, multiorgan dysfunction, and death. The absence of effective therapies has prompted the use of COVID-19 convalescent plasma (CCP) because of the historical efficacy of convalescent plasma treatment in human respiratory viral infections (3–5). Although early CCP treatment of hospitalized patients with COVID-19 reduced mortality in matched-control studies (6–9), randomized clinical trials have yielded mixed results, reducing mortality in one study (10) but not in others (11–16) despite showing signals of efficacy in subgroups.

With a population of just over 500,000 inhabitants, and neighboring Brazil, where high COVID-19 disease incidence was reported, Suriname was confronted with a second COVID-19 wave at the end of May 2020. Given the limited treatment options, we initiated a clinical trial to evaluate clinical efficacy of CCP treatment in patients admitted to the intensive care unit (ICU) with severe or life-threatening COVID-19 in Suriname (SurCovid trial) (17). Because of the low-resource setting and lack of conventional plasmapheresis machines, the HemoClear gravity-driven blood filter was used for CCP production (18) (Video S1). Here, we present the results of CCP treatment on the primary outcome of mortality in COVID-19 ICU patients in Suriname during the second wave of the COVID-19 pandemic.

10.1128/mbio.03379-22.1 VIDEO S1 HemoClear plasma collection instructions. Infographic video illustration of the method for obtaining COVID-19 convalescent plasma using the HemoClear device. Download Movie S1, MP4 file, 3.6 MB. Copyright © 2023 Bihariesingh-Sanchit et al. 2023 Bihariesingh-Sanchit et al. https://creativecommons.org/licenses/by/4.0/ This content is distributed under the terms of the Creative Commons Attribution 4.0 International license.

## RESULTS

### Participants.

A total of 200 patients were enrolled in the study, 58 of which (29%) received CCP. The mean patient age was 51.6 ± 13.7 years, and 110 patients (55%) were male. Patient demographic, clinical and hematological characteristics are described in Table 1. The intervention (CCP) and control (SOC) treatment groups were not significantly different for the vast majority of baseline parameters (demographics, presence of symptoms, comorbidities, medication, and hematological parameters; see Table 1). The CCP and SOC treatment groups did show significant differences in body mass index (BMI; median 32 (27 to 36) versus 28 (26 to 34), respectively, Mann-Whitney test *P* = 0.0338) and muscle pain (53% versus 33%, respectively, Fisher’s exact test *P* = 0.0102). In both cases, the values for the CCP treatment group were less favorable than those for the SOC group. The CCP and SOC treatment groups did not show significant differences for baseline hospital parameters such as days of symptoms, days in hospital until ICU admission, or days of illness until start of treatment.

**TABLE 1:** Demographic and clinical characteristics of the patients at baseline^*a*^

Variable	Control (*n* = 142)	CCP (*n* = 58)	*P*
Demographics			
Age (yr)	52 (14.0)	50 (13.1)	0.3773^*b*^
Mean (SD)			0.6413^*c*^
Cutoff, *n* (%)			
18–50 yr	63 (44.4)	28 (48.3)	
>50 yr	79 (55.6)	30 (51.7)	
Male sex, *n* (%)	79 (55.6)	31 (53.4)	0.8756^*c*^
Race or ethnic group, *n* (%)			0.3655^*d*^
Javanese	42 (29.6)	20 (34.5)	
Maroon	21 (14.8)	9 (15.5)	
Hindustani	29 (20.4)	17 (29.3)	
Creole	25 (17.6)	9 (15.5)	
Amerindian	11 (7.7)	1 (1.7)	
Mix	13 (9.2)	2 (3.4)	
Other	1 (0.7)	0 (0)	
BMI			
Median (IQR)	28 (25.7–33.8)	32 (26.9–36.3)	**0.0338** ^*e*^
Cutoff, *n* (%)			**0.0052** ^*c*^
≤30	85 (59.9)	22 (37.9)	
>30	57 (40.1)	36 (62.1)	
COVID classification severe, *n* (%)	142 (100%)	58 100%	NA
Presence of symptoms, *n* (%)			
Fever	113 (79.6)	47 (82.5)	0.6976^*c*^
Shortness of breath	129 (90.8)	53 (93.0)	0.7825^*c*^
Cough	102 (71.8)	40 (70.2)	1.0000^*c*^
Sputum	24 (17.0)	9 (15.8)	1.0000^*c*^
Loss of taste	29 (20.6)	11 (19.3)	1.0000^*c*^
Loss of smell	20 (14.2)	9 (15.8)	0.8252^*c*^
Itching eyes	3 (2.1)	2 (3.5)	0.6269^*c*^
Red eyes	2 (1.4)	1 (1.8)	1.0000^*c*^
Diarrhea	30 (21.3)	14 (24.6)	0.7060^*c*^
Fatigue	46 (32.6)	27 (47.4)	0.0729^*c*^
Muscle pain	46 (32.6)	30 (52.6)	**0.0102** ^*c*^
Malaise	23 (23.7)	3 (18.8)	1.0000^*c*^
Acute tubular necrosis	24 (17.1)	10 (18.2)	0.8369^*c*^
Vomiting	19 (13.6)	7 (12.3)	1.0000^*c*^
Sore throat	12 (18.6)	5 (10.6)	1.0000^*c*^
Rhabdomyolysis	2 (1.4)	2 (3.5)	0.5808^*c*^
Arrhythmias	6 (4.3)	3 (5.3)	0.7198^*c*^
Time intervals at baseline			
Days hospital until ICU, median (IQR)	1.0 (0–3)	1.0 (0–5)	0.2671^*e*^
Days symptom onset until ICU, median (IQR)	6.0 (3–8)	6.0 (4–8)	0.8065^*e*^
Days illness until treatment start, median (IQR)	4.0 (2–7)	5.0 (3–8)	0.2735^*e*^
Days illness until treatment start cutoff, *n* (%)			0.1683^*c*^
≤7 days	116 (82.9)	42 (73.7)	
>7 days	24 (17.1)	15 (26.3)	
Comorbidities, *n* (%)			
Diabetes mellitus	38 (27.0)	19 (33.3)	0.3894^*c*^
Hypertension	59 (42.1)	26 (45.6)	0.7513^*c*^
Stroke	5 (3.6)	1 (1.8)	0.6746^*c*^
Chronic kidney disease	17 (12.1)	7 (12.5)	1.0000^*c*^
Ischemic heart disease	6 (4.4)	5 (8.8)	0.3051^*c*^
Drug usage, *n* (%)			
Oral diabetics	34 (24.1)	17 (30.4)	0.3727^*c*^
Insulin	8 (5.7)	4 (7.0)	0.7472^*c*^
ACE inhibitors	22 (15.7)	12 (21.1)	0.4076^*c*^
Angiotensin receptor blockers	4 (2.9)	3 (5.3)	0.4169^*c*^
Hematological parameters			
Creatin kinase (U/L), median (IQR)	207 (87.3–489)	191 (110–611)	0.6628^*e*^
Hemoglobin (mmol/L), median (IQR)	7.6 (6.6–8.2)	7.6 (6.5–82)	0.9973^*e*^
Hematocrit (%), mean (SD)	35 (5.5)	36 (5.6)	0.2646^*b*^
Leukocytes (10^9^/L), median (IQR)	11.0 (7.4–16.2)	9.9 (6.9–14.9)	0.3114^*e*^
Thrombocytes (10^9^/L), median (IQR)	217 (170–302)	214 (174–294)	0.5623^*e*^
Lymphocytes (10^9^/L), median (IQR)	1.8 (0.9–5.5)	1.1 (0.9–3.1)	0.1704^*e*^
D-dimer (mg/L), median (IQR)	376 (157–899)	406 (195–1,045)	0.5548^*e*^
Fibrinogen (g/L), mean (SD)	5.2 (1.56)	5.7 (1.79)	0.0627^*b*^
APTT (s), median (IQR)	35.8 (31.9–41.0)	36.9 (30.4–41.3)	0.6429^*e*^
INR, median (IQR)	0.96 (0.90–1.05)	0.96 (0.9–1.04)	0.6506^*e*^
Urea, median (IQR)	8.2 (4.3–17.1)	7.2 (4.0–11.3)	0.2893^*e*^
Creatinine (mol/L), median (IQR)	104 (74.3–201.8)	93 (63.8–129.8)	0.0942^*e*^
Creatinine			0.1120^*c*^
Cutoff, *n* (%)			0.1120^*c*^
≤110 mol/L	79 (55.6)	40 (69.0)	
>110 mol/L	63 (44.4)	18 (31.0)	
Aspartate aminotransferase (U/L), median (IQR)	46 (34–78)	49 (32–67)	0.5014^*e*^
Alanine aminotransferase (U/L), median (IQR)	40 (25–58)	37 (26–48)	0.3705^*e*^
Lactate dehydrogenase (U/L), median (IQR)	421 (296–589)	400 (285–547)	0.7824^*e*^
C-reactive protein (mg/L)			
Median (IQR)	15.5 (8.4–21.8)	16.1 (8.9–26.1)	0.4932^*e*^
Cutoff, *n* (%)			0.6083^*c*^
≤10	43 (30.5)	15 (26.3)	
>10	98 (69.5)	42 (63.7)	
Ferritin (mcg/L)			
Median (IQR)	984 (638–>1,500)	1,111 (551–>1,500)	0.6008^*e*^
Cutoff, *n* (%)			0.8307^*c*^
≤500 μg/L	21 (15.3)	10 (17.2)	
>500 μg/L	116 (84.7)	48 (82.8)	

a CCP, COVID convalescent plasma; SD, standard deviation; IQR, interquartile range. *P* values of <0.05 are in bold.

b Unpaired *t* test.

c Fisher’s exact test.

d Pearson’s chi-square test.

e Mann-Whitney test.

### Primary outcome: mortality.

CCP treatment was associated with significantly fewer deaths: 12 of 58 (21%) patients in the CCP treatment group died compared to 56 of 142 (39%) patients in the SOC group (Fisher’s exact *P = *0.0133) (see Table 2). The number of days spent in the hospital or ICU was significantly higher for the CCP treatment group. These results confirm the protective effect previously reported for the pre-planned interim analysis of this trial (17).

**TABLE 2:** Disease severity in hospitalized patients^*a*^

Variable	Control (*n* = 142)	CCP (*n* = 58)	*P*
Primary outcome, *n* (%)			
Death	56 (39.4)	12 (20.7)	**0.0133** ^*b*^
Secondary outcomes, median (IQR)			
ICU days	5.0 (3–8)	7.0 (5–11)	**0.0023** ^*c*^
Hospital days	11.0 (7–18)	14.0 (10–19)	**0.0309** ^*c*^
Days nasal cannula since ICU admission	5.0 (4–9)	7.0 (4–11)	0.2261^*c*^
Delta CXR	−1.00 (−3.0−1.0)	–3.00 (−4.0–1.0)	**0.0004** ^*c*^
Delta PFR	34.5 (−3.3-92.0)	83.0 (8.3–140.4)	**0.0234** ^*c*^

a CCP, COVID convalescent plasma; IQR, interquartile range. *P* values of <0.05 are in bold.

b Fisher’s exact test.

c Mann-Whitney test.

### Univariate hazard analyses.

**FIG 1:**
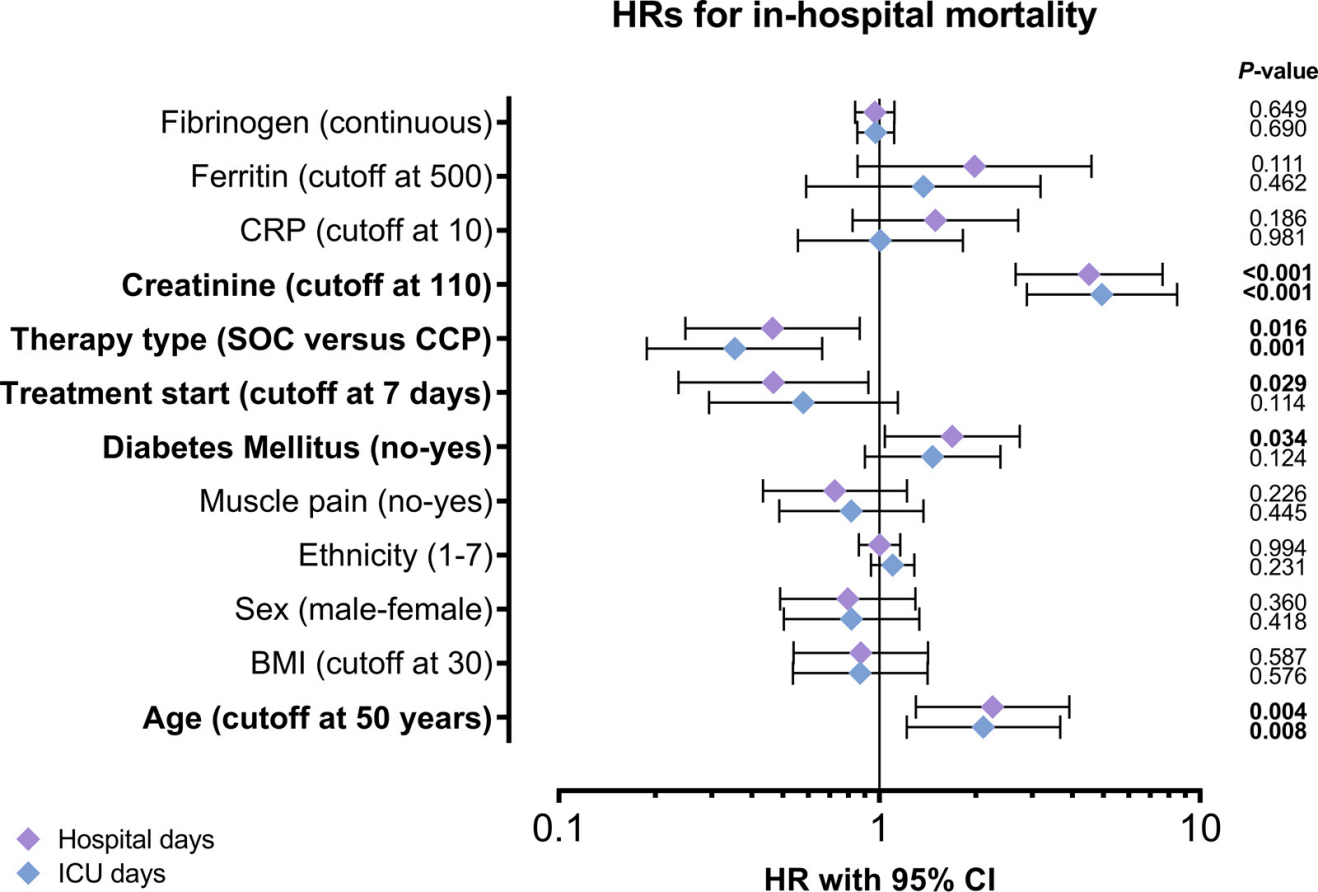
Identification of covariates to be included in multivariate analyses. Univariate Cox proportional analyses of hospital days (purple diamonds) and intensive care unit (ICU) days (blue diamonds) were performed for factors which might have affected in-hospital mortality. Graph depicts hazard ratios with 95% confidence intervals (CI). Log-rank *P* values are shown. Variables in bold were significantly associated with death and included in the multivariate Cox proportional hazard analyses.

In addition to differences identified between the groups at baseline (BMI and muscle pain), other factors which may affect in-hospital mortality have been reported, such as age, sex, ethnicity, diabetes mellitus status, timing of CCP intervention, and levels of creatinine, C-reactive protein, ferritin, and fibrinogen. The effects these factors may have on the primary outcome were evaluated using univariate hazard analyses (Table 3 and Fig. 1). The possible confounders BMI and muscle pain (see Table 1) did not affect hazard ratios and were not included in subsequent multivariate analyses. Using number of ICU days as marker, in addition to treatment type (CCP versus SOC), age and creatinine levels significantly affected hazard ratios (see Table 3): treatment type HR, 0.35 (95% CI, 0.19 to 0.66; *P = *0.001); age HR, 2.11 (95% CI 1.22 to 3.66; *P = *0.008); and creatinine HR, 4.95 (95% CI, 2.88 to 8.48; *P* < 0.001). Using number of hospital days as marker, in addition to treatment type, age, diabetes mellitus status, timing of treatment start and creatinine levels significantly affected hazard ratios (see Table 3): treatment type HR, 0.46 (95% CI, 0.25 to 0.87; *P* = 0.016); age HR, 2.26 (95% CI, 1.30-3.91; *P* = 0.004); diabetes mellitus HR, 1.69 (95% CI,1.04-2.74; *P* = 0.034); treatment start HR, 0.47 (95% CI, 0.24 to 0.92; *P* = 0.029); and creatinine HR, 4.51 (95% CI, 2.66-7.64; *P < *0.001). Next, these factors were included in the multivariate analyses.

**TABLE 3:** Univariate hazard ratios for death^*a*^

Characteristic	ICU days^*c*^			Hospital days^*d*^		
	HR [exp(B)]	95% CI	*P*	HR [exp(B)]	95% CI	*P*
Age (cutoff at 50)	2.110	1.216–3.663	**0.008**	2.256	1.300–3.913	**0.004**
BMI (cutoff at 30)	0.871	0.537–1.413	0.576	0.875	0.540–1.417	0.587
Sex (male-female)	0.818	0.503–1.331	0.418	0.797	0.490–1.296	0.360
Ethnicity (1–7)^*b*^	1.100	0.941–1.284	0.231	1.001	0.862–1.162	0.994
Muscle pain (no or yes)	0.817	0.487–1.372	0.445	0.726	0.433–1.219	0.226
Diabetes mellitus (no or yes)	1.465	0.901–2.383	0.124	1.687	1.039–2.737	**0.034**
Treatment start (cutoff at 7)	0.579	0.294–1.141	0.114	0.467	0.236–0.924	**0.029**
Therapy type (SOC or CCP)	0.354	0.188–0.663	**0.001**	0.464	0.248–0.868	**0.016**
Creatinine (cutoff at 110)	4.945	2.884–8.479	**<0.001**	4.506	2.658–7.640	**<0.001**
CRP (cutoff at 10)	1.007	0.557–1.821	0.981	1.494	0.824–2.708	0.186
Ferritin (cutoff at 500)	1.372	0.591–3.183	0.462	1.980	0.855–4.589	0.111
Fibrinogen (continuous)	0.973	0.853–1.111	0.690	0.968	0.840–1.114	0.649

a ICU, intensive care unit; HR, hazard ratio; CI, confidence interval; SOC, standard of care; CCP, COVID convalescent plasma. *P* values of <0.05 are in bold.

b Ethnicity: 1, Javanese; 2, Maroon; 3, Hindustani; 4, Creole; 5, Amerindian; 6, Mix; 7, Other.

c 28-day follow-up.

d 35-day follow-up.

### Multivariate hazard analysis.

**FIG 2:**
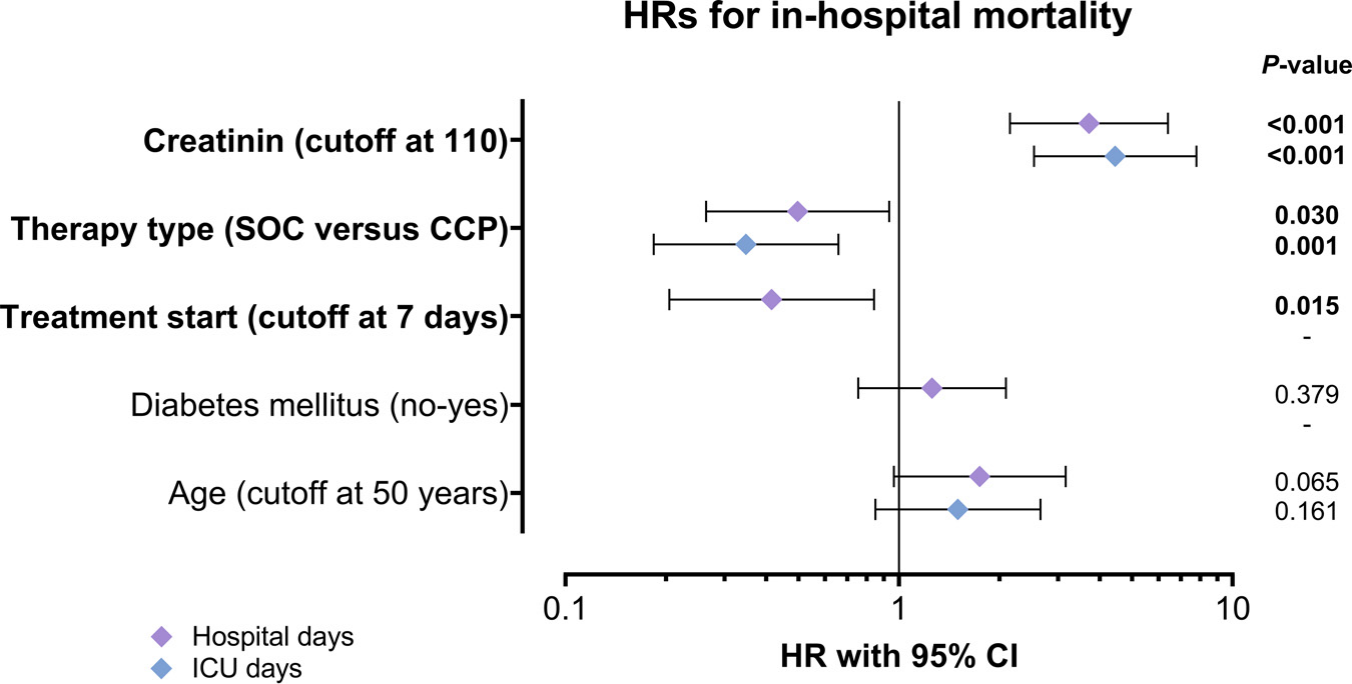
Multivariate analyses of covariates for in-hospital mortality. A multivariate Cox proportional hazard analysis was performed with the univariately significant variables for hospital days (purple diamonds) and ICU days (blue diamonds). Graph depicts hazard ratios with 95% CI. Log-rank *P* values are shown.

In a covariates-adjusted Cox model using the number of ICU days, convalescent plasma transfusion was significantly associated with improved survival (HR, 0.35; 95% CI, 0.18 to 0.66; *P* = 0.001), independent of creatinine levels (HR, 4.45; 95% CI, 2.54 to 7.80; *P < *0.001) (Table 4 and Fig. 2). Similarly, in the model using hospital days, convalescent plasma transfusion was significantly associated with improved survival (HR, 0.50; 95% CI, 0.26 to 0.94; *P* = 0.030), independent of creatinine levels (HR, 3.71; 95% CI, 2.15 to 6.41; *P < *0.001) and timing of treatment start (HR, 0.42; 95% CI, 0.21 to 0.84; *P* = 0.015) (Table 4 and Fig. 2).

**TABLE 4:** Multivariate hazard ratios for death^*a*^

Factor	ICU days^*b*^			Hospital days^*c*^		
	HR [exp(B)]	95% CI	*P*	HR [exp(B)]	95% CI	*P*
Age (cutoff at 50)	1.503	0.850–2.659	0.161	1.747	0.966–3.162	0.065
Diabetes mellitus (no or yes)				1.257	0.755–2.094	0.379
Treatment start (cutoff at 7)				0.415	0.205–0.842	**0.015**
Therapy type (SOC or CCP)	0.348	0.184–0.659	**0.001**	0.497	0.264–0.936	**0.030**
Creatinine (cutoff at 110)	4.450	2.540–7.795	**<0.001**	3.714	2.152–6.410	**<0.001**

a ICU, intensive care unit; HR, hazard ratio; CI, confidence interval; SOC, standard of care; CCP, COVID convalescent plasma. *P* values of <0.05 are in bold.

b 28-day follow-up.

c 35-day follow-up.

### Survival analyses.

**FIG 3:**
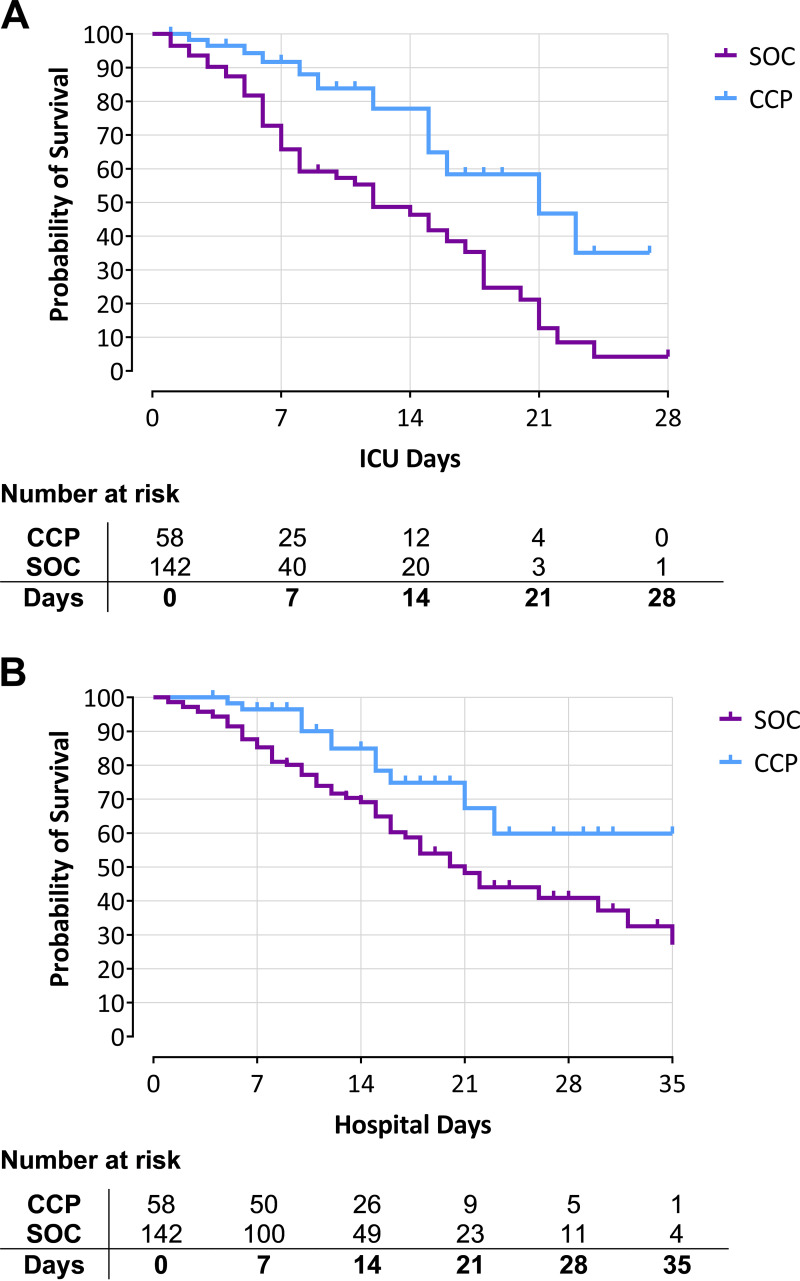
Survival probability during hospital admission for CCP and SOC treatment groups. (A) Kaplan-Meier curve of ICU days for CCP and SOC treatment groups. Right-censoring took place when a person was dismissed from the ICU before the last measured time point; death was counted as an event. Log-rank (Mantel-Cox) *P* < 0.001. The longest ICU admission duration was 27 days for the CCP group, while it was 50 days for the SOC group. (B) Kaplan-Meier curve of hospital days for CCP and SOC treatment groups. Right-censoring took place when a person was dismissed from hospital before the last measured time point; death was counted as an event. Log-rank (Mantel-Cox) *P* = 0.013. The longest hospital admission duration was 37 days in the CCP group and 62 days in the SOC group. Survival analysis was cut off when there were ≤1 patients at risk remaining in at least one of the two groups, at 28 ICU days and at 35 hospital days, respectively.

By the end of the study, 21% of the CCP treatment group and 39% of the SOC treatment group had died (Table 2). The median ICU follow-up time was 7 days (range: 1 to 27) for the CCP treatment group and 5 days (range: 0 to 50) for the SOC treatment group. Median hospital follow-up times were 14 days (range: 4 to 37) and 11 days (range: 1 to 62), respectively. Using either ICU days or hospital days, survival probability was significantly greater in the CCP group than in the SOC treatment group (Fig. 3). Without covariate adjustment, the survival benefit using ICU days had a Cox hazard ratio of 0.35 (0.19 to 0.66) with a log-rank (Mantel-Cox) *P* value of 0.0005 (Fig. 3A). Similarly, using hospital days, the survival benefit had a Cox hazard ratio of 0.46 (0.25 to 0.87) with a log-rank (Mantel-Cox) *P* value of 0.0127 (Fig. 3B).

### Secondary outcomes: CXR score and P/F ratio.

**FIG 4:**
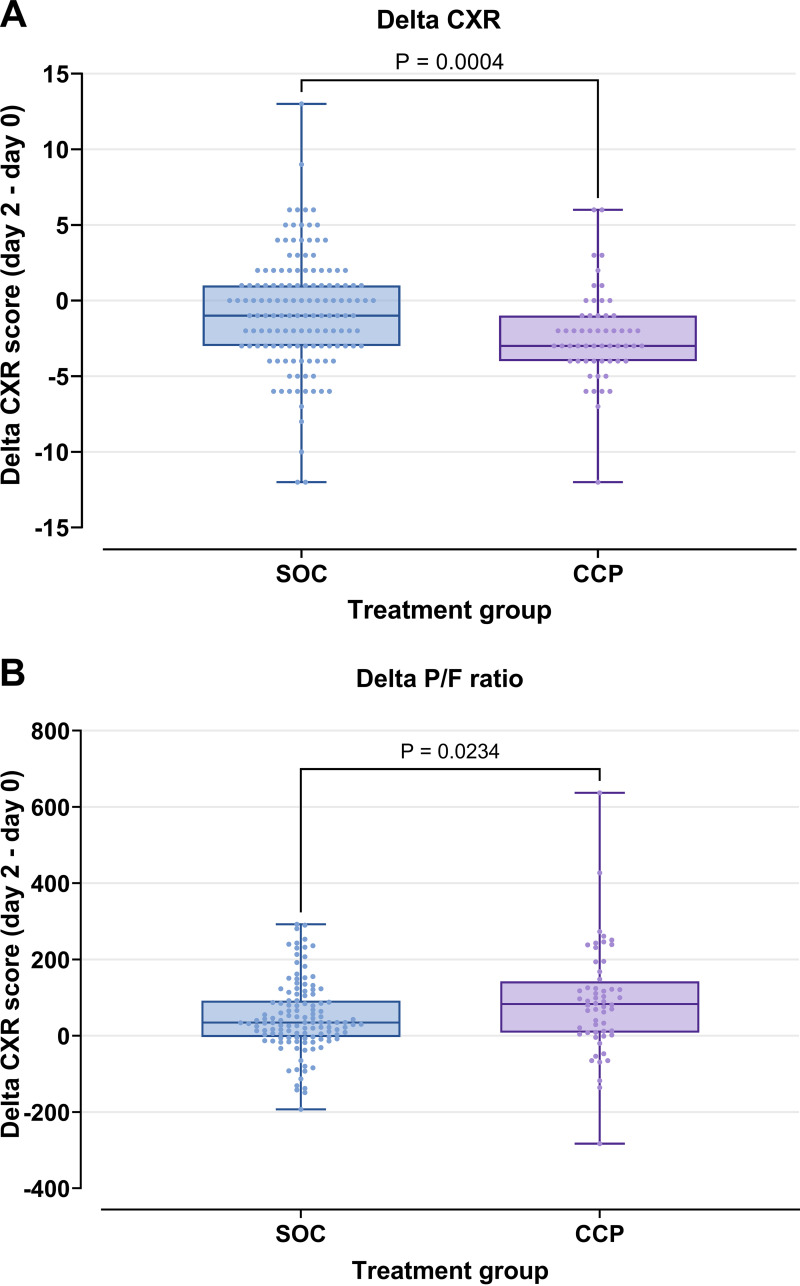
Chest radiographic findings (CXR) score and pulmonary oxygen exchange capacity (P/F) ratio improvement in CCP and SOC treatment groups. The delta CXR and delta PFR were calculated by subtracting the respective scores on day 0 from those on day 2. Graphs show minimum, median, interquartile range, and maximum for each group. (A) The CCP group showed significantly greater improvement in CXR score than the SOC group (Mann-Whitney test). (B) The CCP group showed significantly greater improvement in P/F ratio than the SOC group (Mann-Whitney test).

**(i) Chest radiographic findings (CXR score).** To identify early changes in clinical response, the CXR score was determined for each patient upon ICU admission (day 0) and 48 h after treatment initiation (day 2). The difference in CXR score between day 0 and day 2 was calculated (delta CXR) and compared between the two groups (Fig. 4A). The delta CXR after CCP treatment (median −3 points, interquartile rang [IQR] −4 to −1) was significantly greater than that in the SOC group (median −1 point, IQR −3 to 1; Mann-Whitney *P* = 0.0004) (Table 2).

**(ii) Pulmonary oxygen exchange capacity (P/F ratio).** The P/F ratio was also determined for each patient upon ICU admission and 48 h after treatment initiation. The difference in P/F ratio between days 0 and 2 was calculated (delta PFR) and compared between the two groups (Fig. 4B). The delta PFR after CCP treatment (median 83, IQR 8 to 140) was significantly greater than that in the SOC group (median 35, IQR −3 to 92; Mann-Whitney *P* = 0.0234) (Table 2).

## DISCUSSION

This study compares mortality and other endpoints between ICU COVID-19 patients treated with convalescent plasma plus standard of care (CCP) and a control group of patients hospitalized in the same medical ICU facility treated with standard of care alone (SOC) in a LMIC setting. It demonstrates a significant survival improvement in CCP recipients (HR, 0.35; 95% CI, 0.19 to 0.66; *P* = 0.001).

Early in the pandemic, Duan et al. (19) reported the potential beneficial effect of CCP, showing improvements in respiratory and chest X-ray parameters without serious side effects. Improved ICU survival was also reported in this early phase of the pandemic (20). Limited treatment options and the absence of vaccines at that time made CCP an attractive alternative. Several ICU outcome studies followed with similar results: e.g., Tworek et al. (21) reported a >50% reduction in mortality in the CCP group for a propensity-score matched case control study in Poland, an effect size similar to that of age and timing of treatment initiation. Equivalent mortality rates were reported for patients in Wuhan (22), indicating comparable ICU treatment levels between LMIC versus high-income countries (HIC) with limited resources and capacity (23).

Liu et al. (7) showed that CCP was beneficial in patients with severe or life-threatening COVID-19 disease. These CCP patients experienced a significant improvement of clinical symptoms—such as a reduction of oxygen requirement ratio—as well as improved survival over SOC patients (HR, 0.34; 95% CI, 0.13 to 0.89; *P* = 0.027). But other studies showed limited or even negative results with CCP. Altuntas et al. (24) matched a large retrospective cohort of >1,600 ICU patients with or without CPP treatment, showing an improvement of clinical symptoms but only a modest effect on mortality (24.7% in the CP group, 27.7% in the control group). In one of the rare randomized studies, Simonovich et al. (12) could not detect significant differences in mortality rate between CCP-treated patients and those who received a placebo. However, mortality rates in their SOC and CCP treatment groups were 11.4% and 11.0%, respectively. In addition, most patients were enrolled from the hospital ward (73.3% and 65.8%, respectively) instead of including ICU-admitted patients only. This may well explain the low mortality rate compared to our patient cohort, indicating a possible hurdle to detecting the benefit of CCP treatment in their study population.

The failure of CCP treatment to improve disease outcome in modestly ill patients in the multicenter randomized trial reported by Agarwal et al. (13) severely affected acceptance of CCP treatment and led to its ban in India. This CCP treatment failure was correlated with the use of low-titer CCP, which has since been confirmed in multiple studies. Moreover, studies from the United States showed that high-titer CCP and early treatment initiation resulted in improved mortality reduction (25–27). However, also in the United States, the use of CCP as an emergency drug was restricted and limited to immunocompromised patients only (27). Despite these conflicting results, the promising results from our initial study (17) led us to continue evaluating CPP treatment in ICU patients. This study indeed confirmed the efficacy of CCP treatment in our setting.

The efficacy of CCP treatment is also affected by the source donors and methods of collection/production, which are not standardized and therefore do not guarantee its antiviral contents (28, 29). This might be one of the reasons for the high efficacy of the CCP used in our setting: our CCP was donated by donors early after their recovery and collected using an innovative blood separator, HemoClear, which allows higher particle content of plasma (18), including platelets, exosomes, and high-molecular-weight protein complexes, each of which may have had immune-modulatory and protective effects (30, 31).

Another advantage of the HemoClear device is its reduced cost compared to conventional plasmapheresis machines. While the costs for patient identification, contact, and antibody testing are the same for both methods, initial investment (around $25,000 per machine) and technical staff requirements are higher with conventional plasmapheresis (32). Moreover, due to the 60- to 90-min processing time, each plasmapheresis machine is limited to four collections daily, thus limiting sites in their ability to adjust CCP production to local need. In the United States, federal contracts worth $646 million were paid to U.S. blood centers to collect 500,000 units of COVID convalescent plasma, a unit cost of $1,300 for the U.S. government (33). Due to the added complexity of CCP, blood centers have been reimbursed $600 to $800 per unit (33). In contrast, the HemoClear method costs were well below $300 per unit of CCP.

The availability of CCP is not only a problem in LMICs, but also in HICs. In the nationwide U.S. CCP program, over 30% of the indications were not met due to lack of CCP availability. In addition, CCP programs interfered with conventional blood donation programs because CCP donors were not available for whole-blood donation for 3 months afterwards (34).

Early treatment (within 7 days) after illness onset is paramount in order to treat patients effectively, and this was confirmed in our setting. In order to prevent ICU admission, CCP treatment can even be initiated before ICU admission. Indeed, Libster et al. (35) showed a 48% reduction in hospital admission when CCP was used as treatment within 3 days of becoming ill at home. With bedside production of CCP in primary health care facilities in local communities, not limited by the upfront costs and technological barriers of conventional plasmapheresis, CCP produced by the HemoClear method could be a first line of defense against new virus mutants that are not responsive to existing vaccines, not only in HICs but also in LMICs (36–38).

The initial lack of prophylactic or therapeutic treatment options, combined with limited ICU or even hospital capacity, resulted in decisions to lock down society to reduce viral spread at the start of the pandemic. Decisions not to implement lockdowns, e.g., in Brazil and Tanzania, resulted in excessive deaths and the breakdown of the health care system (39–41). Even in these extreme situations, CCP treatment may help reduce health care burden and disease mortality.

Although this is an exploratory study, it clearly shows the benefit of using the HemoClear-produced CCP in ICU patients in the Suriname LMIC setting. Additional studies can further substantiate our findings and their applicability to both LMIC and HIC and the use of CCP to combat new viral pandemics.

## MATERIALS AND METHODS

This work is reported in adherence to the preferred Strengthening the Reporting of Observational Studies in Epidemiology (STROBE) guidelines. This prospective cohort study was performed at the intensive care unit (ICU) of Academic Hospital Paramaribo and the Wanica Regional Hospital, Suriname, from June 2020 to October 2021 (ISRCTN18304314, https://doi.org/10.1186/ISRCTN18304314). Two hundred patients were included.

### Trial design and oversight.

**FIG 5:**
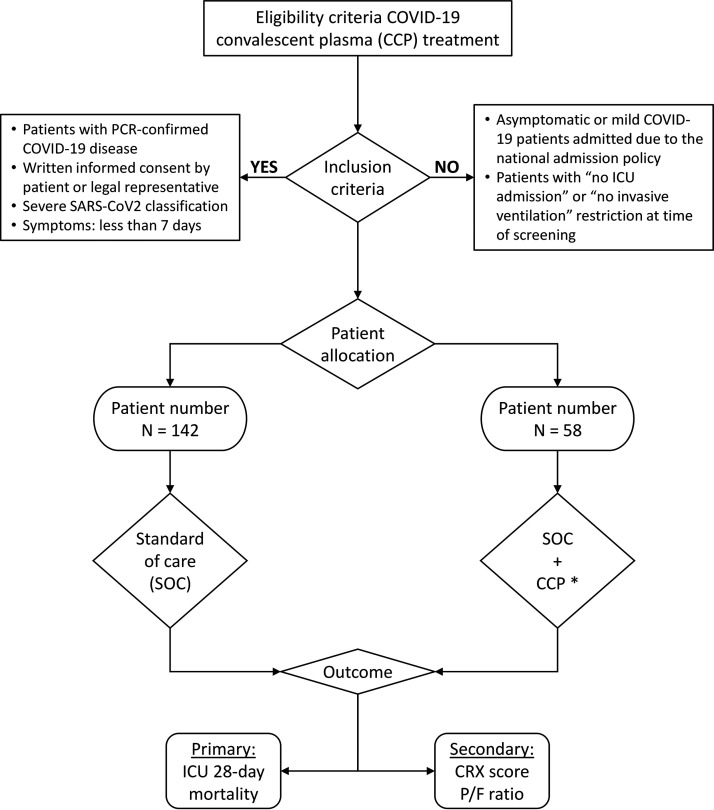
Patient selection flowchart with study enrollment and design. Standard of care treatment group (SOC): oral or intravenous dexamethasone once daily. COVID convalescent plasma group (CCP): 2 units of COVID-19 convalescent plasma. Each unit was infused over a period of at least 30 min, with close observation for the entire duration. Adverse events were medicated (anaphylaxis, antihistamines, corticosteroids, and epinephrine) within reach.

In this prospective cohort study, we compared CCP treatment combined with standard of care versus standard of care alone in patients with severe COVID-19 admitted to the ICU. After referral to the ICU, patients were treated with additional respiratory and circulatory support. After being found eligible for additional CCP treatment, a selection of either standard of care treatment including dexamethasone or standard treatment combined with CCP was initiated. The flow chart shown in Fig. 5 illustrates the study enrollment and design.

Ethical approval was granted by the Suriname Ministry of Health’s Ethics Review Board (registration no. IGAP02-482020; ISRCTN18304314). The data used to support the findings of this study are available from the corresponding author upon request.

### Patient population.

Consenting adult patients (>18 years) with severe COVID-19 were enrolled in the trial in the period from June 2020 until October 2021. The eligibility criteria included written informed consent given by the patient or next of kin, a PCR-confirmed diagnosis of COVID-19, and admittance to the ICU due to progressive respiratory failure ranging between severe and life-threatening acute respiratory distress syndrome based on the Berlin classification (42). For the interventional CCP group, all patients admitted at the ICU who met the inclusion criteria were approached, and 58 patients who consented to CCP infusion plus standard-of-care therapy were recruited to this arm. The control group with standard-of-care therapy alone (SOC treatment group) included patients who did not consent to CCP infusion (*n* = 142). To account for major confounding factors, the following variables were used: age, gender, and comorbidities, including a history of diabetes mellitus, hypertension, drugs, symptoms, and signs (Table 1). In addition, both the SOC and CCP treatment groups received the same standard-of-care concurrent treatment, which included once-daily administration of oral or intravenous dexamethasone for up to 10 days. CCP treatment group patients were infused with two units of 220 mL CCP. For plasma selection, ABO compatibility was considered, regardless of Rhesus factor status. CCP recipients were monitored for serious adverse effects of CCP transfusion, including anaphylaxis. Time of death was recorded as long as patients were in the hospital (either in the ICU or in the regular care ward), with discharge equivalent to survival. For ICU days, the final observation was made at 50 days after the start of treatment; for hospital days, the last observation was at 62 days after the start of treatment.

Convalescent plasma donor recruitment, plasma preparation, and data collection were performed as described previously (17).

### Statistical analysis.

Unless stated otherwise, all analyses were performed on the complete data set. General descriptive statistics were assessed using IBM SPSS Statistics for Windows (version 29.0.0.0). Differences between the CCP and SOC treatment groups were analyzed with Pearson chi-square or Fisher’s exact tests (where suitable) for categorical variables, *t* tests for parametric continuous variables, and Mann-Whitney tests for nonparametric continuous variables. Differences in outcome measures were analyzed using univariate and multivariate Cox proportional hazard analyses. The mortality risk was assessed using Kaplan Meier survival analysis and hazard ratios were calculated with a Cox proportional hazard model. CXR scores and P/F ratios were summarized by representing the median and spread in a boxplot, and differences between the CCP and SOC treatment groups were analyzed with Mann-Whitney tests. A *P* value of 0.05 was considered to represent a significant difference. Graphs of study data were generated using GraphPad Prism (version 9.4.1).
